# *N*-Butyl-4-methyl-6-phenyl­pyrimidin-2-amine

**DOI:** 10.1107/S1600536810009384

**Published:** 2010-03-20

**Authors:** Hoong-Kun Fun, Wan-Sin Loh, Anita Hazra, Shyamaprosad Goswami

**Affiliations:** a X-ray Crystallography Unit, School of Physics, Universiti Sains Malaysia, 11800 USM, Penang, Malaysia; b Department of Chemistry, Bengal Engineering and Science University, Shibpur, Howrah 711 103, India

## Abstract

In the title compound, C_15_H_19_N_3_, the pyrimidine ring is approximately planar [maximum deviation = 0.007 (1) Å] and forms a dihedral angle of 3.15 (6)° with the benzene ring. In the crystal packing, inter­molecular N—H⋯N hydrogen bonds link pairs of neighbouring mol­ecules into dimers with *R*_2_^2^(8) ring motifs. These dimers are stacked along the *b* axis.

## Related literature

For the biological importance of substituted amino pyrimidines, see: Katrizky (1982 ▶); Brown & Lyall (1964 ▶); Jonckers *et al.* (2001 ▶). For their synthesis by microwave processes, see: Goswami *et al.* (2009 ▶). For a related structure, see: Fun *et al.* (2006 ▶). For hydrogen-bond motifs, see: Bernstein *et al.* (1995 ▶). For bond-length data, see: Allen *et al.* (1987 ▶). For the stability of the temperature controller used for the data collection, see: Cosier & Glazer (1986 ▶).
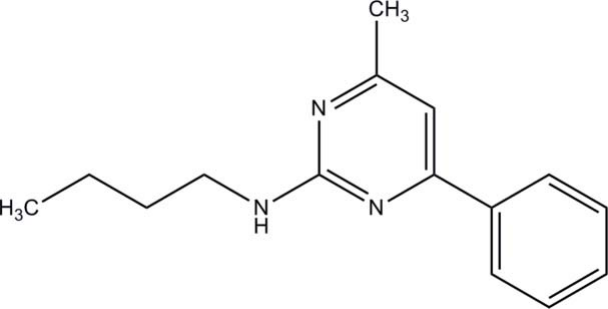


## Experimental

### 

#### Crystal data

C_15_H_19_N_3_
*M* *_r_* = 241.33
Monoclinic, 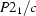

*a* = 13.4828 (9) Å
*b* = 5.1618 (3) Å
*c* = 22.8462 (11) Å
β = 123.863 (3)°
*V* = 1320.29 (13) Å^3^
*Z* = 4
Mo *K*α radiation
μ = 0.07 mm^−1^
*T* = 100 K
0.30 × 0.23 × 0.08 mm

#### Data collection

Bruker SMART APEX DUO CCD area-detector diffractometer
Absorption correction: multi-scan (*SADABS*; Bruker, 2009 ▶) *T*_min_ = 0.978, *T*_max_ = 0.994
13801 measured reflections
3829 independent reflections
3085 reflections with *I* > 2σ(*I*)
*R*_int_ = 0.031

#### Refinement

*R*[*F* ^2^ > 2σ(*F* ^2^)] = 0.044
*wR*(*F* ^2^) = 0.170
*S* = 1.15
3829 reflections
169 parameters
H atoms treated by a mixture of independent and constrained refinement
Δρ_max_ = 0.54 e Å^−3^
Δρ_min_ = −0.28 e Å^−3^

### 

Data collection: *APEX2* (Bruker, 2009 ▶); cell refinement: *SAINT* (Bruker, 2009 ▶); data reduction: *SAINT*; program(s) used to solve structure: *SHELXTL* (Sheldrick, 2008 ▶); program(s) used to refine structure: *SHELXTL*; molecular graphics: *SHELXTL*; software used to prepare material for publication: *SHELXTL* and *PLATON* (Spek, 2009 ▶).

## Supplementary Material

Crystal structure: contains datablocks global, I. DOI: 10.1107/S1600536810009384/sj2745sup1.cif

Structure factors: contains datablocks I. DOI: 10.1107/S1600536810009384/sj2745Isup2.hkl

Additional supplementary materials:  crystallographic information; 3D view; checkCIF report

## Figures and Tables

**Table 1:** Hydrogen-bond geometry (Å, °)

*D*—H⋯*A*	*D*—H	H⋯*A*	*D*⋯*A*	*D*—H⋯*A*
N3—H3*B*⋯N2^i^	0.798 (17)	2.283 (17)	3.0802 (14)	177 (2)

Symmetry code: (i) 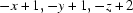.

**Fig. 1.**
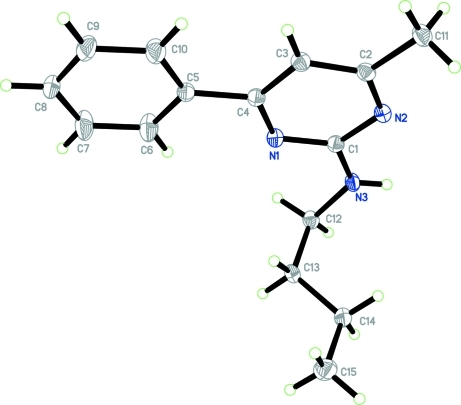
The molecular structure of the title compound, showing 50% probability displacement ellipsoids and the atom-numbering scheme.

**Fig. 2.**
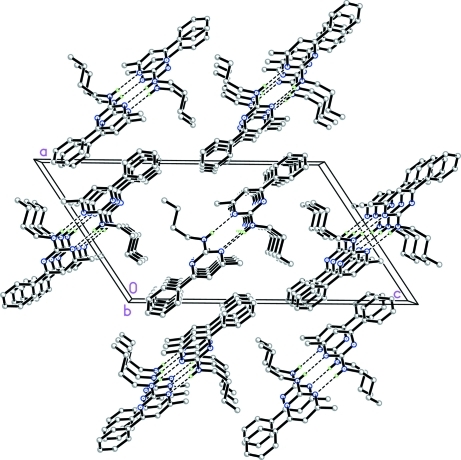
The crystal packing of the title compound, viewed along the b axis, showing the R22(8) ring motifs. H atoms not involved in the intermolecular interactions (dashed lines) have been omitted for clarity.

## supplementary crystallographic information

### Comment

Substituted amino pyrimidines are highly biologically important molecules (Katrizky, 1982; Brown & Lyall, 1964; Jonckers *et al.*, 2001). Recently we have synthesized various substituted amino pyrimidines by microwave process (Goswami *et al.*, 2009). Here we report the crystal structure of 2-butylamino-4-methyl-6-phenylpyrimidine.

In the title compound (Fig. 1), the pyrimidine ring (C1/N2/C2/C3/C4/N1) is approximately planar with a maximum deviation of 0.007 (1) Å at atom N2 and forms a dihedral angle of 3.15 (6)° with the benzene ring (C5–C10). The bond lengths are within normal values (Allen *et al.*, 1987) and similar to those in the crystal structure of 4,6-diphenylpyrimidin-2-ylamine (Fun *et al.*, 2006).

In the crystal packing (Fig. 2), two neighbouring molecules are linked by intermolecular N3—H3B···N2 hydrogen bonds (Table 1) to form dimers with *R*_2_^2^(8) ring motifs (Bernstein *et al.*, 1995). These dimers are stacked along the *b* axis.

### Experimental

A mixture of *S*-methylisothiourea sulphate (556 mg, 2.0 mmol), potassium carbonate (345 mg, 2.5 mmol) and butylamine (292 mg, 4.0 mmol) was thoroughly mixed together and then irradiated at 450 Watt for 12 min in a microwave oven. The solid mass was washed with CHCl_3_ to remove the unreacted butylamine and it was then dried. The solid residue formed was mixed with benzoylacetone (648 mg, 4.0 mmol) and again irradiated at 300 Watt for 5 min. Then it was dissolved in water and extracted with chloroform. The crude product was purified by column chromatography (silica gel, 100-200 mesh) with 15% ethyl acetate in petroleum ether as eluant. Single crystals were grown by slow evaporation of a chloroform solution. Yield: 75 %; *Mp*: 328-329 K.

### Refinement

H3B was located in a difference Fourier map and refined freely [N–H = 0.797 (18) Å]. The remaining H atoms were positioned geometrically and refined using a riding model with *U*_iso_(H) = x*U*_eq_(C), where x = 1.5 for methyl H and 1.2 for all other H atoms [C–H = 0.93 to 0.97 Å]. A rotating group model was applied to the methyl groups.

### Crystal data

**Table:**

C_15_H_19_N_3_	*F*(000) = 520
*M**_r_* = 241.33	*D*_x_ = 1.214 Mg m^−^^3^
Monoclinic, *P*2_1_/*c*	Mo *K*α radiation, λ = 0.71073 Å
Hall symbol: -P 2ybc	Cell parameters from 4691 reflections
*a* = 13.4828 (9) Å	θ = 3.0–30.0°
*b* = 5.1618 (3) Å	µ = 0.07 mm^−^^1^
*c* = 22.8462 (11) Å	*T* = 100 K
β = 123.863 (3)°	Plate, colourless
*V* = 1320.29 (13) Å^3^	0.30 × 0.23 × 0.08 mm
*Z* = 4	

### Data collection

**Table:**

Bruker SMART APEX DUO CCD area-detector diffractometer	3829 independent reflections
Radiation source: fine-focus sealed tube	3085 reflections with *I* > 2σ(*I*)
graphite	*R*_int_ = 0.031
φ and ω scans	θ_max_ = 30.0°, θ_min_ = 1.9°
Absorption correction: multi-scan (*SADABS*; Bruker, 2009)	*h* = −18→18
*T*_min_ = 0.978, *T*_max_ = 0.994	*k* = −7→7
13801 measured reflections	*l* = −31→31

### Refinement

**Table:**

Refinement on *F*^2^	Primary atom site location: structure-invariant direct methods
Least-squares matrix: full	Secondary atom site location: difference Fourier map
*R*[*F*^2^ > 2σ(*F*^2^)] = 0.044	Hydrogen site location: inferred from neighbouring sites
*wR*(*F*^2^) = 0.170	H atoms treated by a mixture of independent and constrained refinement
*S* = 1.15	*w* = 1/[σ^2^(*F*_o_^2^) + (0.0985*P*)^2^ + 0.2466*P*] where *P* = (*F*_o_^2^ + 2*F*_c_^2^)/3
3829 reflections	(Δ/σ)_max_ = 0.001
169 parameters	Δρ_max_ = 0.54 e Å^−^^3^
0 restraints	Δρ_min_ = −0.28 e Å^−^^3^

### Special details

**Table:**

Experimental. The crystal was placed in the cold stream of an Oxford Cryosystems Cobra open-flow nitrogen cryostat (Cosier & Glazer, 1986) operating at 100.0 (1) K.
Geometry. All esds (except the esd in the dihedral angle between two l.s. planes) are estimated using the full covariance matrix. The cell esds are taken into account individually in the estimation of esds in distances, angles and torsion angles; correlations between esds in cell parameters are only used when they are defined by crystal symmetry. An approximate (isotropic) treatment of cell esds is used for estimating esds involving l.s. planes.
Refinement. Refinement of *F*^2^ against ALL reflections. The weighted *R*-factor wR and goodness of fit *S* are based on *F*^2^, conventional *R*-factors *R* are based on *F*, with *F* set to zero for negative *F*^2^. The threshold expression of *F*^2^ > σ(*F*^2^) is used only for calculating *R*-factors(gt) etc. and is not relevant to the choice of reflections for refinement. *R*-factors based on *F*^2^ are statistically about twice as large as those based on *F*, and *R*- factors based on ALL data will be even larger.

### Fractional atomic coordinates and isotropic or equivalent isotropic displacement parameters (Å^2^)

**Table:**

	*x*	*y*	*z*	*U*_iso_*/*U*_eq_	
N1	0.28959 (9)	0.3936 (2)	0.81414 (5)	0.0154 (2)	
N2	0.37437 (9)	0.2822 (2)	0.93559 (5)	0.0152 (2)	
N3	0.45114 (9)	0.6155 (2)	0.90539 (5)	0.0157 (2)	
C1	0.36872 (10)	0.4257 (2)	0.88392 (6)	0.0144 (2)	
C2	0.29524 (10)	0.0896 (2)	0.91422 (6)	0.0156 (2)	
C3	0.20994 (10)	0.0413 (2)	0.84312 (6)	0.0165 (2)	
H3A	0.1549	−0.0926	0.8290	0.020*	
C4	0.20988 (10)	0.2004 (2)	0.79384 (6)	0.0147 (2)	
C5	0.12498 (10)	0.1654 (2)	0.71633 (6)	0.0163 (2)	
C6	0.13025 (13)	0.3317 (3)	0.67022 (7)	0.0273 (3)	
H6A	0.1849	0.4674	0.6881	0.033*	
C7	0.05468 (14)	0.2972 (3)	0.59776 (7)	0.0319 (3)	
H7A	0.0593	0.4097	0.5676	0.038*	
C8	−0.02738 (12)	0.0971 (3)	0.57018 (7)	0.0239 (3)	
H8A	−0.0776	0.0737	0.5217	0.029*	
C9	−0.03385 (13)	−0.0677 (3)	0.61560 (7)	0.0295 (3)	
H9A	−0.0893	−0.2019	0.5975	0.035*	
C10	0.04193 (13)	−0.0343 (3)	0.68824 (7)	0.0271 (3)	
H10A	0.0369	−0.1470	0.7183	0.033*	
C11	0.30354 (12)	−0.0752 (3)	0.97066 (6)	0.0199 (3)	
H11A	0.3830	−0.1431	1.0003	0.030*	
H11B	0.2477	−0.2157	0.9494	0.030*	
H11C	0.2852	0.0276	0.9985	0.030*	
C12	0.46551 (11)	0.7606 (2)	0.85587 (6)	0.0167 (2)	
H12A	0.5112	0.9164	0.8787	0.020*	
H12B	0.3875	0.8127	0.8160	0.020*	
C13	0.52875 (11)	0.6029 (2)	0.82927 (6)	0.0176 (3)	
H13A	0.4836	0.4455	0.8075	0.021*	
H13B	0.5286	0.7020	0.7931	0.021*	
C14	0.65693 (11)	0.5300 (3)	0.88617 (6)	0.0194 (3)	
H14A	0.6596	0.4587	0.9263	0.023*	
H14B	0.7060	0.6848	0.9018	0.023*	
C15	0.70840 (12)	0.3326 (3)	0.86024 (7)	0.0266 (3)	
H15A	0.7902	0.2983	0.8970	0.040*	
H15B	0.7040	0.4004	0.8197	0.040*	
H15C	0.6633	0.1747	0.8477	0.040*	
H3B	0.4963 (16)	0.637 (4)	0.9468 (9)	0.026 (4)*	

### Atomic displacement parameters (Å^2^)

**Table:**

	*U*^11^	*U*^22^	*U*^33^	*U*^12^	*U*^13^	*U*^23^
N1	0.0164 (5)	0.0151 (5)	0.0137 (4)	−0.0001 (3)	0.0077 (4)	−0.0003 (4)
N2	0.0164 (5)	0.0158 (5)	0.0136 (4)	0.0003 (3)	0.0084 (4)	0.0003 (4)
N3	0.0175 (5)	0.0173 (5)	0.0112 (4)	−0.0035 (4)	0.0073 (4)	−0.0013 (4)
C1	0.0149 (5)	0.0140 (5)	0.0145 (5)	0.0006 (4)	0.0084 (4)	−0.0006 (4)
C2	0.0170 (5)	0.0154 (5)	0.0164 (5)	0.0016 (4)	0.0106 (5)	0.0006 (4)
C3	0.0175 (5)	0.0154 (5)	0.0172 (5)	−0.0013 (4)	0.0101 (4)	−0.0002 (4)
C4	0.0149 (5)	0.0144 (5)	0.0148 (5)	0.0016 (4)	0.0084 (4)	−0.0005 (4)
C5	0.0153 (5)	0.0180 (6)	0.0149 (5)	0.0016 (4)	0.0080 (4)	−0.0005 (4)
C6	0.0324 (7)	0.0241 (7)	0.0168 (6)	−0.0091 (6)	0.0084 (5)	0.0008 (5)
C7	0.0376 (8)	0.0336 (8)	0.0155 (6)	−0.0093 (6)	0.0093 (6)	0.0024 (5)
C8	0.0183 (6)	0.0332 (7)	0.0140 (5)	−0.0009 (5)	0.0050 (5)	−0.0032 (5)
C9	0.0244 (7)	0.0385 (8)	0.0193 (6)	−0.0157 (6)	0.0082 (5)	−0.0066 (6)
C10	0.0275 (7)	0.0329 (8)	0.0179 (6)	−0.0126 (6)	0.0107 (5)	−0.0022 (5)
C11	0.0233 (6)	0.0201 (6)	0.0168 (5)	−0.0024 (5)	0.0115 (5)	0.0021 (4)
C12	0.0190 (5)	0.0156 (5)	0.0156 (5)	−0.0008 (4)	0.0097 (4)	0.0019 (4)
C13	0.0192 (6)	0.0207 (6)	0.0132 (5)	0.0000 (4)	0.0093 (5)	0.0012 (4)
C14	0.0188 (6)	0.0227 (6)	0.0156 (5)	0.0016 (4)	0.0090 (5)	−0.0001 (4)
C15	0.0234 (6)	0.0283 (7)	0.0270 (7)	0.0025 (5)	0.0133 (5)	−0.0050 (5)

### Geometric parameters (Å, °)

**Table:**

N1—C4	1.3453 (15)	C8—H8A	0.9300
N1—C1	1.3459 (15)	C9—C10	1.3921 (18)
N2—C2	1.3362 (15)	C9—H9A	0.9300
N2—C1	1.3593 (15)	C10—H10A	0.9300
N3—C1	1.3520 (15)	C11—H11A	0.9600
N3—C12	1.4570 (15)	C11—H11B	0.9600
N3—H3B	0.797 (18)	C11—H11C	0.9600
C2—C3	1.3934 (16)	C12—C13	1.5284 (16)
C2—C11	1.4957 (16)	C12—H12A	0.9700
C3—C4	1.3932 (16)	C12—H12B	0.9700
C3—H3A	0.9300	C13—C14	1.5214 (17)
C4—C5	1.4899 (16)	C13—H13A	0.9700
C5—C10	1.3886 (18)	C13—H13B	0.9700
C5—C6	1.3910 (18)	C14—C15	1.5263 (18)
C6—C7	1.3894 (18)	C14—H14A	0.9700
C6—H6A	0.9300	C14—H14B	0.9700
C7—C8	1.383 (2)	C15—H15A	0.9600
C7—H7A	0.9300	C15—H15B	0.9600
C8—C9	1.382 (2)	C15—H15C	0.9600
			
C4—N1—C1	116.94 (10)	C5—C10—H10A	119.7
C2—N2—C1	116.17 (10)	C9—C10—H10A	119.7
C1—N3—C12	121.92 (10)	C2—C11—H11A	109.5
C1—N3—H3B	117.5 (13)	C2—C11—H11B	109.5
C12—N3—H3B	120.3 (13)	H11A—C11—H11B	109.5
N1—C1—N3	117.88 (10)	C2—C11—H11C	109.5
N1—C1—N2	125.85 (10)	H11A—C11—H11C	109.5
N3—C1—N2	116.27 (10)	H11B—C11—H11C	109.5
N2—C2—C3	122.10 (10)	N3—C12—C13	112.34 (10)
N2—C2—C11	116.56 (10)	N3—C12—H12A	109.1
C3—C2—C11	121.33 (11)	C13—C12—H12A	109.1
C4—C3—C2	117.72 (11)	N3—C12—H12B	109.1
C4—C3—H3A	121.1	C13—C12—H12B	109.1
C2—C3—H3A	121.1	H12A—C12—H12B	107.9
N1—C4—C3	121.19 (11)	C14—C13—C12	114.32 (10)
N1—C4—C5	115.89 (10)	C14—C13—H13A	108.7
C3—C4—C5	122.91 (11)	C12—C13—H13A	108.7
C10—C5—C6	118.46 (11)	C14—C13—H13B	108.7
C10—C5—C4	121.79 (11)	C12—C13—H13B	108.7
C6—C5—C4	119.73 (11)	H13A—C13—H13B	107.6
C7—C6—C5	120.68 (13)	C13—C14—C15	112.29 (10)
C7—C6—H6A	119.7	C13—C14—H14A	109.1
C5—C6—H6A	119.7	C15—C14—H14A	109.1
C8—C7—C6	120.54 (13)	C13—C14—H14B	109.1
C8—C7—H7A	119.7	C15—C14—H14B	109.1
C6—C7—H7A	119.7	H14A—C14—H14B	107.9
C9—C8—C7	119.15 (12)	C14—C15—H15A	109.5
C9—C8—H8A	120.4	C14—C15—H15B	109.5
C7—C8—H8A	120.4	H15A—C15—H15B	109.5
C8—C9—C10	120.51 (13)	C14—C15—H15C	109.5
C8—C9—H9A	119.7	H15A—C15—H15C	109.5
C10—C9—H9A	119.7	H15B—C15—H15C	109.5
C5—C10—C9	120.66 (13)		

### Hydrogen-bond geometry (Å, °)

**Table:**

*D*—H···*A*	*D*—H	H···*A*	*D*···*A*	*D*—H···*A*
N3—H3B···N2^i^	0.798 (17)	2.283 (17)	3.0802 (14)	177 (2)

Symmetry codes: (i) −*x*+1, −*y*+1, −*z*+2.
